# Argon: Systematic Review on Neuro- and Organoprotective Properties of an “Inert” Gas

**DOI:** 10.3390/ijms151018175

**Published:** 2014-10-10

**Authors:** Anke Höllig, Anita Schug, Astrid V. Fahlenkamp, Rolf Rossaint, Mark Coburn, Argon Organo-Protective Network (AON)

**Affiliations:** 1 Department of Neurosurgery, University RWTH Aachen, 52074 Aachen, Germany; E-Mails: ahoellig@ukaachen.de (A.H.); anschug@ukaachen.de (A.S.); 2 Department of Anesthesiology, University RWTH Aachen, 52074 Aachen, Germany; E-Mails: afahlenkamp@ukaachen.de (A.V.F.); rrosaint@ukaachen.de (R.R.)

**Keywords:** argon, neuroprotection, organoprotection, inert gas, hypoxia, ischemia, cytoprotection

## Abstract

Argon belongs to the group of noble gases, which are regarded as chemically inert. Astonishingly some of these gases exert biological properties and during the last decades more and more reports demonstrated neuroprotective and organoprotective effects. Recent studies predominately use *in vivo* or *in vitro* models for ischemic pathologies to investigate the effect of argon treatment. Promising data has been published concerning pathologies like cerebral ischemia, traumatic brain injury and hypoxic ischemic encephalopathy. However, models applied and administration of the therapeutic gas vary. Here we provide a systematic review to summarize the available data on argon’s neuro- and organoprotective effects and discuss its possible mechanism of action. We aim to provide a summary to allow further studies with a more homogeneous setting to investigate possible clinical applications of argon.

## 1. Introduction

Argon belongs to the noble gases and generally is regarded as an inert, non-reactive element. Even its name (from the Greek “αργός”—inert) refers to its chemical inactivity. In fact, biological effects of the noble gases including argon have been identified starting in the 1930s: its narcotic properties under hyperbaric circumstances were described beginning with studies investigating argon as a possible breathing gas for divers [1]. Recently, neuroprotective and organoprotective features have been identified [2,3,4,5,6].

In general, most promising therapeutics—especially neuroprotectants—identified through preclinical studies have failed to demonstrate efficacy in clinical trials due to heterogeneous experimental settings, inadequate sample sizes, inappropriate time and dosage of application and so on [7,8]. Concerning argon’s beneficial properties, most of the evidence has been accomplished by *in vitro*, *in vivo* and rarely human studies. Again, the multitude of anecdotal reports and experimental models applied hinders the overall assessment of argon’s therapeutic potential but also its possible side effects. Therefore we performed a systematic review on the current literature on argon. We provide an overview of available data on argon’s organoprotective and particularly its neuroprotective features as well as potential side effects. Further, we illustrate the current data on the possible mechanism of action and future perspectives for therapeutic applications of argon.

## 2. Results

**Figure 1:**
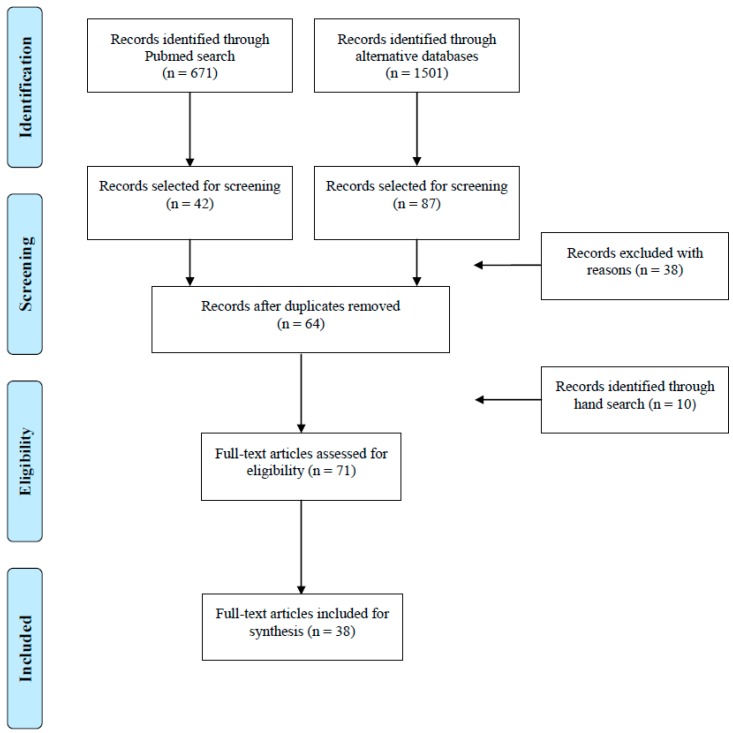
Diagram showing literature search procedure and results.

The PubMed search revealed 671 hits, from which 42 records were identified as relevant for screening. The alternative databases (Embase, Scisearch, Biosys, gms) presented 1501 records using the same search strategy. Eighty-seven records were regarded relevant. Thirty-five articles had to be excluded with regard to content (review articles, comments or articles on technical applications of argon, abstracts and poster presentations); one article had to be rejected as only available in the Chinese language. Duplicates (*n* = 65) among the two database searches were eliminated. In Figure 1 the procedure is summarized. In total, 38 relevant full text articles were identified. Eleven out of 38 (29%) studies were conducted before, and 27 (71%) after the year 2000. Human studies are scarce (*n* = 6, see Table 1) and most of them had been motivated by technical considerations in the context of diving or aerospace. *In vivo* animal experiments dealing with the effects of argon are much more common (*n* = 22, summarized in Table 2) and the number of publications on *in vivo* data has increased recently (16 out of 22 articles have been published later than 2000). Most animal experiments were carried out with rats (16 out of 22); in two studies, Japanese quail eggs were used. *In vitro* studies are dominated by the use of murine organotypic brain slices (4 out of 10 studies; see Table 3).

Comparisons with other noble gases were drawn in 7 (out of 22) animal studies and 5 *in vitro* studies. Frequently the effect of argon was compared to that of helium (*n* = 7) and xenon (*n* = 5).

In human studies, descriptions of argon’s narcotic effect and the possible increase of resistance against hypoxia were most common, whereas among *in vivo* animal studies, the neuroprotective or organoprotective properties of argon were the main topic (11 out of 22). In general most of the studies dealt with argon’s narcotic effect and the reaction of organisms to hypoxia under argon atmosphere (*n* = 14). Notably most of these studies were carried out before the year 2000 (9 out of 14). Neuroprotection and organoprotection are relatively new topics: All of the studies covering these topics (*n* = 17) were carried out after the year 2000. Neuroprotection is, with 11 articles, the field of interest most frequently highlighted in the recent years. Besides tissue protection, recent studies often dealt with the identification of argon’s mechanism of action. In total, 11 investigations addressed this question with 10 of them carried out after 2000. Most studies concerning protective effects of argon and its mechanism of action were carried out using animal or *in vitro* models.

Notably, argon failed to show protective properties in two studies [9,10], whereas other studies on tissue protection could only demonstrate a partial benefit of argon treatment (*i.e.*, only functional improvement or benefit under certain circumstances like timing of applications) [11,12].

## 3. Discussion 

Our systematic review has highlighted various studies on argon’s effects applying heterogeneous models and questions. We will discuss similarities and differences of the approaches and results.

### 3.1. Physiological Studies

The first descriptions of argon’s biological effects arose in the context of diving medicine. Mental impairment at high pressures had been observed. Behnke and Yarbrough in 1939 tried to elucidate the role of argon in producing narcotic effects in humans [1]. The first physiological data (like respiratory resistance) could be assessed. Further studies were carried out evaluating mental performance and subjective rating of condition as measurement of narcosis with 80% argon and 20% oxygen under different pressure levels [13]. In humans, long-term effects (up to 7 days) under hyperbaric argon atmosphere were examined demonstrating improved work performance and a shift in lipid metabolism. An increased resistance to hypoxic hypoxia under argon atmosphere was suggested [14]. These results were confirmed by a study testing human oxygen consumption during physical exercise breathing a gas mix with 30% argon. An increase of oxygen consumption under argon was observed, therefore a catalytic activity of argon on oxygen kinetics was supposed [15]. Another long-term study carried out for 9 days (14% oxygen, 33% nitrogen, 54% argon and 0.2% carbon dioxide for 6 days followed by 10% oxygen, 35% nitrogen, 55% argon and 0.2% carbon dioxide) demonstrated no detrimental influence on work performance [16].

Narcotic potency was also examined in mice [9] and rats [17,18]. Therefore metabolism, oxygen consumption and resistance towards hypoxia under argon (different species and organ slices) were investigated [9,19,20,21]. A favorable metabolic condition with a distinct energy metabolism and elevated oxygen consumption was supposed, thus resulting in increased resistance towards hypoxia [20,22]. Furthermore, an improved survival of animals under hypoxic atmosphere was demonstrated [23,20], whereas an earlier series of experiments with white mice did not indicate a beneficial effect of argon on survival [9]. Under hypoxic atmosphere containing argon a change in development (faster development and less teratogenic pathologies) was observed, which also has been attributed to the change of metabolism under argon [19,24,25].

In conclusion, argon seems to improve resistance towards hypoxia. Metabolic changes, cell membrane dependent mechanisms, recovery of mitochondrial enzymes and oxygen synergism have been discussed to explain this phenomenon. Studies concerning mechanism of action will be discussed hereafter in more detail.

**Table 1:** Human studies.

Experimental Model	Number of Cases	Dose and Concentration	Outcome Parameter	Results of Experiments	Conclusion	Reference
Mental performance in Ar-N_2_-O_2_-atmosphere	*n* = 4, male	six days (5 m depths): 14% O_2_, 33% N_2_, 54% Ar, 0.2% CO_2_ followed by three days: 10% O_2_, 35% N_2_, 55% Ar, 0.2% CO_2_	Adaptive biocontrol of cortical (ABC) bioelectric activity synchronization, emotional and mental performance (Luscher test), “Minesweeper” and “Tetris” performance	Partial improvement of performance, overall no decrease of ABC skill	Despite fluctuations of anxiety levels no influence on work performance, tendency to loose preservation of adaptation process with argon-mix	Antonov & Ershova (2009) [16]
Assessment of mental impairment breathing argon at different pressures (corresponding to 90–130 m diving depth)	*n* = 4	69% Ar, 11% N_2_, 20% O_2_, duration not specified	Self-assessment of diving depth	No effect on mental status for normobaric argon, mental impairment at pressure levels corresponding to depths of 90–130 m (tendency to overestimate diving depth)	Narcotic effect of argon is greater than that of nitrogen	Behnke & Yarbrough (1939) [1]
Comparison of argon and nitrogen narcosis at 1 to 10 ATA (0.1 resp. 1.1 MPa)	*n* = 10	80% Ar, 20% O_2_ or air (different pressure levels)	Assessment of narcosis: mental arithmetic, subjective estimate of narcosis, adjective checklist.	Arithmetic: numbers of errors increase with high pressure (with argon mix more than with air), subjective rating of narcosis: increases with higher pressure (with argon mix more than with air), adjective checklist: number of responses increases with pressure (highly variable)	Inert gases exert qualitavely identical effects	Fowler & Ackles (1972) [13]
(a) Exposition to white noise (85 dB) for 1 h; (b) Exposition of rats to hypoxic gas mix; (c) Exposition of hair cells (*ex vivo*) to hypoxic Ar-/N_2_-saturated medium	*n* = 10	(a) 24% Ar, 60% N_2_, 16% O_2_, normobaric, duration not specified; (b) ≥25% Ar, 4%–5% O_2_ normobaric; (c) 95% Ar, 5% CO_2_ or 95% N_2_, 5% CO_2_	(a) Pure-tone audiometry, TEOAE, DPOAE, BERA, EcohG; (b) Survivability of rats; (c) Survival time of hair cells in medium	(a) Improved condition of acoustic system in the argon treated group; (b) Increased survival in Ar-gas mix; (c) Increased survival of hair cells in Ar-containing medium	Oto- and neuroprotective effect of argon, attenuates effects of hypoxia	Matsnev *et al*. (2007) [23]
Long term (7 day) effects of hypoxic argon-oxygen mixture on human performance	*n* = 4 male	7 days (10 m depths): 0.2 kg/cm^2^ O_2_, 0.8 kg/cm^2^ N_2_, 1.0 kg/cm^2^ Ar	Assessment of respiratory, cardiovascular and neurological parameters, evaluation of physical and mental work performance	Shift in lipid metabolism, better work performance with hyperbaric 15% Ar-O_2_ mixture	Argon is physiologically active causing increased resistance to hypoxic hypoxia (redox-reaction)	Pavlov *et al*. (1999) [14]
Oxygen consumption breathing Ar-containing gas mixtures during physical (submaximal) exercise	*n* = 7, male	15% O_2_, 30% Ar, 55% N_2_ or 15% O_2_, 85% N_2_	Oxygen consumption, heart rate, ventilation frequency during physical exercise breathing hypoxic gas mixtures	Increase of oxygen consumption during exercise breathing Ar-mix compared to N_2_-mix	Catalytic activity of argon on kinetics of oxygen consumption which might increase tolerance towards hypoxia	Shulagin *et al*. (2001) [15]

Ar = argon; O_2_ = oxygen; N_2_ = nitrogen; CO_2_ = carbon dioxide; TEOAE = transitory evoked otoacoustic emission; DPOAE = distortion product otoacoustic emissions; BERA = brainstem evoked response audiometry; EcohG = electrocochleography.

**Table 2:** Animal experiments.

Experimental Model	Species, Age	Number of Cases	Pressure, Dose and Concentration	Outcome Parameter	Results of Experiments	Conclusion	Reference
Assessment of argon’s narcotic potency after pretreatment with GABA-antagonists (GABA_A_-receptor-antagonist gabazine; GABA_B_-receptor antagonist, GABA_A_-receptor antagonist benzodiazepine site)	Sprague Dawley rats, adult	*n* = 6 per group	Argon was dosed at 0.1 Mpa/min until narcosis was reached	Loss of righting reflex	Increase of argon threshold pressure after pretreatment with GABA_A_-receptor antagonist and GABA_A_-receptor antagonist for benzodiazepine site	Argon may interact directly with the GABA_A_ receptor and partly with its benzodiazepine site	Abraini *et al*. (2003) [26]
Evaluation of relationship of locomotor and motor activity and striatal dopamine release under argon narcosis	Sprague Dawley rats, adult	Total *n* = 108	2 MPa (with 0.1 MPa/min)	Behavioral analysis, quantification of striatal dopamine release	Biphasic pattern with initial hyperactivity after compression; decrease of activity and dopamine release after 1 MPa	Dopamine release could be related to decrease of hyperactivity under argon narcosis	Balon *et al*. (2003) [27]
Assessment of reaction in response to minimal electroshock and antagonisation with antipsychotic drug (Frenquel)	Wistar rats	*n* = 46; 102 experiments	12.6 atm abs (=1.3 MPa)	Reaction to minimal electroshock	Greater narcotic potency of argon compared with nitrogen, partly abolished by Frenquel	Argon narcosis may arise from histotoxic hypoxia; Frenquel somehow decreases the narcotic effect	Bennett (1963) [17]
Cardiac arrest for 7 min followed by 3 min resuscitation (CPR), postconditioning with argon	Sprague Dawley rats, adult	*n* = 7 per group	1 h after CPR: 70% Ar, 30% O_2_ for 1 h	Neurological performance 7d after CPR, hippocampal cell loss	Better neurological performance (NDS score) and less neuronal damage of neocortex and hippocampus (C3/4), no difference in caspase 3/9 expression	Long lasting functional effect paralleled by less neuronal damage C3/4	Brücken *et al*. (2013) [28]
Cardiac arrest for 7 min followed by 3 min resuscitation (CPR), postconditioning with argon, pretreatment with 5HD (K_ATP_-Channel-Blocker)	Sprague Dawley rats, adult	*n* = 9 per group	1 h after CPR: either 70% Ar and 30% O_2_ or 40% Ar, 30% O_2_ and 30% N_2_	Neurological performance 8d after CPR, neuronal loss (neocortex, hippocampal C3/4)	Better neurological performance in argon –treated group (70% Ar > 40% Ar), less neuronal loss (regardless of Ar-concentration), no influence of 5HD on beneficial argon effect	Argon exerts dose dependent neuroprotective effect, K_ATP_-Channels seem not to be involved in the mechanism of action	Brücken *et al*. (2014) [29]
Cardiac arrest for 7 min followed by 3 min resuscitation (CPR), postconditioning with argon	Sprague Dawley rats, adult	*n* = 8 per group	1h of 70% Ar and 30% O_2_ either 1 or 3 h after CPR or no argon treatment	Neurological performance 8d after CPR, neuronal loss (neocortex, hippocampal C3/4, basal ganglia)	Better neurological performance and less neuronal loss in neocortex and hippocamplas C3/4 in both argon—treated groups, less neuronal damage in basal ganglia (3 h delay)	Argon exerts a neuroprotective effect even after treatment delayed for 3 h	Brücken *et al*. (2014) [30]
Assessment of oxygen consumption and development time of different species	Yeast, Drosophila, Mouse, Zootermopsis, Tenebrio, Cnemidophorus, Coloenyx		80% Ar, 20% O_2_	Oxygen consumption of different species, development time of larvae	Argon alters rate of metabolism and development (acceleration of metamorphosis) in some animals	Argon–either at atmospheric or high pressure is not inert	Cook (1950) [19]
(a) OGD (brain slices); (b) NMDA-induced brain damage (*in vivo*); (c) MCAO (*in vivo*)	Sprague Dawley rats, adult	*n* = 8 to *n*= 14 per group	(a) 15%–75% Ar for 3 h after OGD; (b) 15%–75% Ar for 3 h (1 h after NMDA); (c) 50% Ar, 25% N_2_, 25% O_2_ for 3 h (2 h after MCAO)	(a) LDH release after OGD; (b) Extent of brain damage; (c) Neurologic outcome and extent of brain damage	(a) Most pronounced reduction of LDH release compared to N_2_ in 50% argon treated (less with 37.5% and 75% Ar); (b) Significantly attenuation of NMDA induced brain damage with 37.5 and 50% Ar; (c) Reduction of cortical ischemic volume by Ar, increase of subcortical brain damage, decrease of neurological score compared to sham	Argon shows antiexcitotoxic effects (oxygen like properties), but due the demonstrated adverse effects (increase of subcortical damage. and decrease of neurological function in the argon treated group after MCAO) results do not support therapeutic postischemic application of argon, protective effect after NMDA-induced brain injury and OGD.	David *et al*. (2012) [11]
2 h of MCAO, 1 h after MCAO either 50% Ar/50% O_2_ or 50% N_2_/50% O_2_	Sprague-Dawley rats, adult	*n* = 53	50% Ar/50% O_2_ or 50% N_2_/50% O_2_ for 1 h, normobaric	24 h after MCAO, expression analysis of inflammatory and growth factors, cell count of neurons, astrocytes and microglia	In argon-treated MCAO significantly higher expression levels of IL-1beta, IL-6, iNOS, TGF-beta, and NGF were found compared to MCAO. VEGF was significantly elevated compared to sham. Significant reduction of neurons only occurred in the penumbra after MCAO	An elevated expression of several inflammatory and growth factors following MCAO + argon compared to MCAO + placebo and sham	Fahlenkamp *et al*. (2014) [31]
Effect of hypoxic argon containing gas mix (for 4 days) on early embryogenic development	Japanese quail eggs	*n* = 30	15% O_2_, 30% N_2_, 55% Ar or 15% O_2_, 85% N_2_ for 4 days	Assessment of survival and development	With argon containing gas mix up to 60% development, normal morphology, without argon only 17% reached adequate developmental state	Positive effect of argon on embryonic development in hypoxic atmosphere	Gur’eva *et al*. (2008) [24]
Transplantation of harvested kidneys after storage in Ar-, Xe- or N_2_-saturated solution	Wistar rats, adult	*n* = 60	Storage in Ar-, Xe- or N_2_-saturated solution for 6 h	Assessment of renal function (Creatinine clearance, urinary albumin) 7 and 14 days after transplantation, histological examination of transplanted kidneys 14 days after transplantation	Creatinine clearance higher and urinary albumin lower as well as better renal architecture in Ar-treated group compared to N_2_ treated with a more pronounced effect by argon than by xenon treatment	Decrease of ischemia-reperfusion injury, improved graft function and maintained anatomical structure after Ar- treatment (compared to Xe and N_2_)	Irani *et al*. (2011) [32]
LAD occlusion for 30 min, preconditioning with 70% Ar/He/Ne/30% O_2_ or hypoxic preconditioning	New Zealand white, rabbit	*n* = 98	Preconditioning with 3 cycles each 5 min (70% Ar/He/Ne, 30% O_2_), normobaric	Assessment of infarct size compared to hypoxic preconditioning compared to control (no preconditioning)	Significant reduction of infarct size after preconditioning with Ar, He and Ne	More pronounced cardioprotection with Ar-preconditioning compared to hypoxic preconditioning	Pagel *et al*. (2007) [33]
LAD occlusion, cardiac arrest for 8 min, CPR for 5 min followed by defibrillation, postconditioning for 4 h with either Ar/O_2_ or N_2_/O_2._	Domestic pig, male	*n* = 12	70% Ar, 30% O_2_ or 70% N_2_, 30% O_2_ for 4 h, normobaric	Assessment of survival and neurological function 72 h after CPR, serum neuron-specific enolase (NSE) and troponin, Immunohistochemistry of brain slices	Better neurological performance in argon-treated group, significantly lower increase in serum NSE and minimal histological brain injury	Faster, complete neurologic recovery with argon treatment, no detrimental side effects, mainly functional improvement assessed	Ristagno *et al*. (2014) [12]
Narcotic effect of compression in argon atmosphere	Rats, 15 weeks	*n* = 15	Ar 100–800 kPa	Assessment of behavior during compression and decompression	First signs of narcosis from 500 kPa on, subsequently falling asleep at 800 kPa (8 of 10 animals)	Demonstration of narcotic properties of argon	Ružička *et al*. (2007) [18]
2 h of MCAO, 1 h after MCAO either 50% Ar/50% O_2_ or 50% N_2_/50% O_2_	Sprague Dawley rats, adult	*n* = 22	50% Ar/50% O_2_ or 50% N_2_/50% O_2_ for 1 h, normobaric	24 h after MCAO: neurological assessment, evaluation of infarct size	Improved composite adverse outcome, reduction of infarct volume (overall, cortical and subcortical) in argon-treated group	Argon demonstrates *in vivo* neuroprotective properties (reduced infarct size), but no improvement concerning neurological outcome and mortality	Ryang *et al*. (2011) [34]
Survivability of rats in hypoxic argon containing atmosphere	Wistar rats		Hypoxic atmosphere: O_2_ (4%–8%), different concentrations of Ar (0%–80%), N_2_ (15%–87%) and CO_2_ (0%–8%)	Survival rate of rats in hypoxic atmospheres with different gas mix	Adding argon increases survival rate, adding CO_2_ and increasing temperature reduces survival rate	Adding argon improves hypoxic tolerance	Soldatov *et al*. (1998) [20]
Effect of hypoxic environment on development	Japanese quail eggs		10% O_2_, 55% Ar, 35% N_2_ or 10% O_2_, 90% N_2_	Assessment of survival rate and occurrence of teratogenic pathologies	Argon containing gas mixture reduces occurrence of teratogenic events, 100% mortality after 7 days with both mixtures	Argon reduces incidence of teratogenic events probably by stimulation of metabolism	Soldatov *et al*. (2002) [25]
Influence of hypoxic atmosphere (O_2_/Ar or O_2_/N_2_) on brain metabolism	White rats		Hypoxic atmosphere: O_2_ (7%) with Ar or N_2_	Detection of NADH/NAD in brain slices	Argon attenuates hypoxia induced metabolic impairment	Positive effect on cerebral energy metabolism by argon	Vdovin *et al*. (1998) [21]
Decompression in atmospheres containing Ar or He	Male albino mice	Total *n*= 231	79% Ar/ He, 21% O_2_, decompression to 179 mmHg	Survival rate during decompression at different temperatures, assessment of oxygen consumption	Survival rate in argon containing atmosphere similar to air during decompression, higher survival rate in helium containing atmosphere	Helium promotes hypoxic resistance of mice, but none observed for argon	Witherspoon *et al*. (1964) [9]
Hypoxic ischemic brain injury: ligation of right carotid artery, hypoxia (8% O_2_, 92% N_2_) 1h after ligation for 90 min (moderate) or 120 min (severe) followed by postconditioning with Ar/He/Xe or control	Sprague Dawley rats, age: 7 days	*n* = 5 per group	120 min after hypoxia: 70% Ar, 30% O_2_ for 90 min, normobaric	Cell viability after moderate and severe hypoxia (7 and 14 days thereafter), infarct volume, neurologic/motor performance, protein analysis contralateral hemisphere	Improved cell viability with postconditiong (Ar > Xe, He) after moderate hypoxia, improvement after severe hypoxia by Ar and Xe, induction of Bcl-2 (contralateral hemisphere) after Ar-postconditioning, neurologic function in noble gas treated animals better than control	Pronounced neuroprotective effect by argon after mild and severe hypoxia, possibly acts via upregulation of Bcl-2 expression	Zhuang *et al*. (2012) [35]

Ar = argon; N_2_ = nitrogen; O_2_ = oxygen; Xe = xenon; He = helium; Ne = neon; CO_2_ = carbon dioxide; GABA_A_-receptor = gamma-aminobutyric acid A receptor; GABA_B_-receptor = gamma-aminobutyric acid B receptor; Frenquel = γ-pipradol or Azacyclonol; CPR = cardiopulmonary resuscitation; MCAO = middle cerebral artery occlusion; K_ATP_-Channel = ATP-sensitive potassium channel; OGD = oxygen glucose deprivation; NMDA-receptor = *N*-Methyl-d-aspartic acid-receptor; MCAO = middle cerebral artery occlusion; LDH = Lactate dehydrogenase; LAD = left anterior descending artery; Bcl-2 = B-cell lymphoma 2.

**Table 3:** *In vitro* studies.

Experimental Model	Studied Material	Pressure, Dose and Concentration	Outcome Parameter	Results of Experiments	Conclusion	Reference
Evaluation of interaction of argon and tPA (tissue plasminogen activator) on enzymatic and thrombolytic efficiency: catalytic efficiency of tPA, blood clot formation and thrombolysis	Whole blood (Sprague Dawley rats)	25%–75% Ar, 25% O_2_	Catalytic and thrombolytic efficiency of tPA	Concentration dependent dual effect of argon on tPA effect: at concentrations higher than 50% argon increases catalytic and thrombolytic efficiency, but decreases them at concentrations lower 50%	Effect may be due to elastase binding of argon or to its interaction with oxygen competing for tPA binding and overcoming the hypoxic effect with higher concentrations (oxygen synergism)	David *et al*. (2013) [36]
Nitrogen or argon hypoxia (OGD) for 90 min followed by postconditioning with argon or nitrogen (each 75%)	Foetal (18 days) BALB/c mice, brain slices	OGD: 75% Ar, 20% N_2_ or 95% N_2_, 5% CO_2_; followed by: 75% Ar or 75% N_2_, 20% O_2_, 5% CO_2_, normobaric	Cell viability quantified by MTT assay	Neuroprotective effect of argon after OGD (less than Xe, also tested), in the absence of OGD: improved cell viability with argon compared to control (naïve)	Argon shows a significant neuroprotective effect but less pronounced than with xenon	Jawad *et al*. (2009) [37]
Exposure of primary neuronal and astroglial cell cultures and the microglial cell line BV-2 to 50% argon, additionally stimulation of microglia with LPS	BALB/c mice (primary cultures), BV-2 cell line	Exposure of primary cultures to 50% Ar for 15–120 min (*vs*. control N_2_ instead of Ar)	Protein analysis after treatment and stimulation with LPS, analysis of RNA-expression	Increase of ERK 1/2 phosphorylation in microglia by argon (mediated by upstream kinase MEK1/2), no phosphotyrosine phosphatase inhibition, no augmentation of LPS-mediated ERK 1/2 activation, no relevant modification of LPS-induced cytokine expression by argon	Short enhanced activation of ERK1/2 via MEK by argon (in primary cultures and microglia), activation does not take part via interference with phosphotyrosine phosphatases. No substantial modification of cytokine expression after LPS-exposure in microglia	Fahlenkamp *et al*. (2012) [38]
Membrane stability of peritoneal macrophages (mice) under argon or nitrogen saturated medium after UV-induced damage	Peritoneal macrophages (mice)	Normobaric, hypoxic Ar or N_2_ saturated medium	Measurement of intracellular pH, ability to build up fluorescein	Normobaric environment with Ar or N_2_ protects plasmatic membranes from UV-induced damage	Resistance against UV-induced damage is elevated by hypoxic Ar or N_2_ containing environment	Galchuk *et al*. (2001) [39]
(a) *In vitro* traumatic brain injury (hippocampal brain slices), effect of glycine administration; postconditioning with noble gas; (b) Patch clamp study to evaluate receptor effect.	(a) C57BL/6 mice (brain slices); (b) HEK293 cells	Different concentrations	(a) Extent of cell injury after trauma; (b) Effect on NMDA-mediated or TREK-1 currents	(a) Argon at 50% atm shows neuroprotective effects attenuate secondary injury after trauma (but less than xenon), glycine does not reverse argon’s positive effect; (b) NMDA-mediated or Trek-1 currents are not influenced by argon	Argon’s neuroprotective effect seems not to be mediated by NMDA-receptor glycine site, potassium channels neither seem to be involved	Harris *et al*. (2013) [40]
(a) *In vitro* trauma (hippocampal brain slice); (b) OGD for 30 min followed by postconditioning with argon.	Brain slices C57BL/6	Postconditioning with 25%-74% argon (directly after lesion) or with 50% argon up to 3 h delayed	Extent of cell injury 72 h after lesion	(a) Neuroprotective effect of argon after TBI even if applied with delay up to 3 h (most effective at 50% argon concentration); (b) Dose dependent neuroprotective effect of argon after OGD even if applied with delay up to 3 h	Neuroprotective effect of argon in two types of brain lesions, effect even noticeable with 50% argon after delayed application	Loetscher *et al*. (2009) [41]
Oxygen consumption of yeast and liver slices (rat) in inert gas mixture	Yeast, liver slices (Sprague Dawley rats)	20%–80% Ar	Oxygen consumption	Reduced oxygen consumption of yeast and live slices in buffer bubbled with argon, no effect on homogenized liver slices	Depression of oxygen consumption under argon may be due to cell membrane mediated effect as not noticeable with homogenized samples	Maio *et al*. (1967) [42]
Preconditioning with noble gas (75% for 3 h), 24 h thereafter OGD for 3 h	Cultured human renal tubular cells (HEK2)	75% argon, helium, neon, krypton or xenon for 3 h (24 h after injury)	Cell viability 24 h after OGD, without OGD: protein analysis for p-Akt, HIF-1α and Bcl-2	No protection from injury by argon, decrease of HIF-1α with argon	No protective effect with argon (but with xenon), for argon: no influence on Bcl-2 expression and decrease of HIF-1α expression	Rizvi *et al*. (2010) [10]
Effect of gas mixtures on induction of apoptotic cell death (by tyrosine kinase inhibitors, DNA-damaging agents and mitochondrial toxins)	Human osteosarcoma cells (U2OS)	75% Ar or Xe or He or Ne or Kr or N_2_, 20% O_2_, 5% CO_2_	Automated fluorescence microscopy to reveal cell death	Argon (and xenon) prevent cell loss after damaging agents, activation of signal transduction pathway sensitive to Z-VAD-fmk, suppresses pathways of intrinsic apoptosis (cytochrome C, caspase 3)	Argon suppresses multiple manifestations of the intrinsic apoptotic pathway	Spaggiari *et al*. (2013) [43]
Trauma of organotypic cultures (organ of Corti, rat) with (a) hypoxia; (b) cisplatin or gentamycin	Organotypic cultures (organ of Corti), Wistar rat	(a) 95% Ar or N_2_, 5% CO_2_ *vs*. normoxia; (b) 74% Ar or N_2_, 21% O_2_, 5% CO_2_	Assessment of cell viability after 48 h	Lower damage in argon treated group after hypoxia as well as cisplatin or gentamycin damage	Protective effect of argon probably affecting Ca^+^ metabolism	Yarin *et al*. (2005) [44]

Ar = argon; N_2_ = nitrogen; O_2_ = oxygen; Xe = xenon; He = helium; Ne = neon; CO_2_ = carbon dioxide; tPA = tissue plasminogen activator; ERK1/2 = extracellular-signal-regulated kinases 1/2; MEK1/2 = MAPKK = mitogen-activated protein kinase kinase; LPS = lipopolysaccharide; NMDA-receptor = *N*-Methyl-d-aspartic acid-receptor; TREK-1 = Potassium channel subfamily K member 2; p-Akt = phospho-Akt; HIF-1α = hypoxia inducible factor 1α; Bcl-2 = B-cell lymphoma 2; Z-VAD-fmk = pan caspase inhibitor; TBI = traumatic brain injury.

### 3.2. Neuroprotective and Organoprotective Properties

Within a multitude of experimental models protective effects of argon were investigated: *In vitro* mostly fetal organotypic murine brain slices were applied. Lesion was induced either by mechanical trauma (*in vitro* traumatic brain injury-TBI) or by oxygen-glucose-deprivation (OGD) simulating global metabolic stress, *i.e.*, ischemia. Mechanical as well as metabolic stress was diminished by argon application repeatedly [37,40,41,43]. The concentration of argon varied, but averaged at least 50% (in one study 50% atm was applied). Dose dependency for argon treatment after OGD was demonstrated by Loetscher and colleagues, whereas the most effective concentration after *in vitro* TBI was identified with 50% argon. Even delayed application of postconditioning with argon (up to 3 h after injury) still resulted in decrease of cell death compared to controls without argon treatment [41]. Without injuring the brain slices, application of 75% argon was even able to reduce cell death when compared to controls and showed a less pronounced protective effect than xenon [37]. Another organotypic model assessed hypoxic and toxic resistance of hair cells (organ of Corti) under argon containing atmosphere demonstrating an otoprotective effect [44].

*In vivo* the most common models are those inducing hypoxia either resulting in cerebral ischemia (by middle cerebral artery occlusion, or hypoxic ischemic brain injury with ligation of carotid artery and exposure to hypoxia) or myocardial ischemia (by LAD-left anterior descending artery-occlusion) or both (by cardiac arrest (CA) followed by delayed resuscitation (CPR)). In line with the clear protective effect after OGD—an *in vitro* model for cerebral ischemia—Ryang and colleagues [34] demonstrated a reduction of infarct volume and improved composite adverse outcome following argon postconditioning using an MCAO rat model. With the same model (MCAO) but different application time of argon David and colleagues [11] also showed a reduction of cortical infarct volume, but subcortical brain damage increased with argon treatment. In this connection the argon treated animals revealed worse neurological performance (compared to sham), which was found at days 1 and 2 after MCAO. This contrasts with xenon that provides both cortical (greater than argon) and subcortical neuroprotection and further showed improved neurological outcome in the same conditions of MCAO model and timing of treatment [45]. As discussed by David *et al*., differences between their results and those of Ryang *et al*. as regards to subcortical neuroprotection could be due to differences in study protocol, particularly timing of treatment (intraischemic *vs*. postischemic). However, in the same study of David and coworkers, protective effects of argon after OGD were confirmed and, *in vivo*, an attenuation of NMDA-induced brain damage was shown. Neuroprotective properties after hypoxia (hypoxic ischemic brain injury rat model) were confirmed by Zhuang and colleagues [35]. A more pronounced beneficial effect regarding cell viability for postconditioning with argon was described vis-a-vis nitrogen and even xenon. Combining some features of the aforementioned models, some groups use a resuscitation model to induce cerebral ischemia: In pigs and rats postconditioning with 70% of argon resulted in improved neurological outcome [12,28]. The morphological extent of brain damage (at least for some regions) was reduced compared to controls. In rats dose dependency of the beneficial effect after resuscitation was demonstrated with better neurological outcome after treatment with 70% argon than with 40% [29].

Cardioprotective effects with decrease of infarct size were shown by an *in vivo* study using argon as a preconditioning drug with a rabbit model [33]. Another possible application of argon is to protect donor organs before transplantation. Rat kidneys harvested in argon saturated solution demonstrated better functional and morphological condition than controls (nitrogen saturated solution) or even xenon treated group [32].

Finally the only human study on neuroprotection was carried out to investigate the effect of argon treatment on exposure to white noise. Improved condition of the acoustic system was shown assuming an oto- and neuroprotective effect of treatment [23]. This hypothesis was strengthened by experimental data on improved hair cell survival with argon treatment.

In conclusion argon’s neuroprotective and organoprotective properties were confirmed by various studies using a multitude of experimental models primarily to simulate hypoxic and less frequently mechanical cell stress. Neuroprotection is the field most commonly covered and most studies underscore the beneficial effect of argon treatment. Nevertheless, results are biased by heterogeneously applied experimental models and differences in study protocols (different timing, concentration and duration of treatment).

### 3.3. Mechanism of Action

Very little is known about the actual mechanism of action of argon. Abraini and colleagues investigated the involvement of GABA-receptors by examining argon’s narcotic potency in rats after pretreatment with specific GABA-receptor antagonists. They discovered that in a rat model argon threshold pressure had to be increased after pretreatment with GABA_A_-receptor antagonists and to a lesser extent after GABA_A_-receptor antagonists for the benzodiazepine site. This was not the case after pretreatment with a GABA_B_-receptor antagonist. Thus—similar to nitrogen—involvement of GABA_A_- and the benzodiazepine site of GABA_A_-receptors, but none of GABA_B_-receptors, was hypothesized [26]. However, this finding is limited by the fact that Abraini and colleagues used the (hyperbaric) narcotic properties of argon as outcome parameter. Therefore it is problematic transferring the results into the area of neuroprotection, which is achieved under normobaric circumstances. Furthermore at atmospheric pressures, argon did not provoke an intracellular acidosis in macrophages that is induced by other benzodiazepine-sensitive GABA_A_-receptor agonists [46]. Thus, two distinct, independent methods of action are conceivable dependent on ambient pressure and response measured.

Another *in vivo* study correlated the extent of striatal dopamine release with the narcotic effect of argon. Decrease of striatal dopamine release was seen in parallel to gas narcosis [27]. Again, this finding relies on argon’s narcotic properties not its cytoprotective properties as indicator of outcome.

Similar to xenon, which inhibits NMDA-receptors [47], this receptor type was investigated during argon treatment. Application of glycine did not reverse the beneficial effect of argon after *in vitro* TBI, therefore involvement of the glycine site of the NMDA-receptor in argon’s mechanism of action was ruled out. Further, using electrophysiology (patch clamp technique) no effect of argon on NMDA-mediated currents was found, likewise for currents flowing through TREK-1, a two-pore-domain potassium channel [40]. In an *in vivo* study (resuscitation rat model), pretreatment with a K_ATP_-channel blocker (5-Hydroxydecanoate = 5HD) also failed to impact argon’s beneficial effect [29]. Therefore, neither NMDA receptors nor potassium channels seem to be involved. However, these results will have to be confirmed in further experiments.

Another *in vivo* study tested in a rat model of hypoxic ischemic brain injury and postconditioning with 70% argon, helium or xenon the expression of three proteins involved in the intrinsic apoptotic pathway: Bax, Bcl-2, and Bcl-xL. Treatment with argon, helium, and xenon increased the expression of Bcl-2. Surprisingly, helium and xenon, with the exception of argon, increased Bcl-xL, a prosurvival protein, whereas expression of Bax, which promotes cell death, was induced after treatment with helium [35]. Again, these results may reflect the uniqueness of each noble gas in regard to its mechanism of action. Further, noble gas modulation of prosurvival proteins has to be elucidated.

Using cultured renal tubular cells (HEK2) prosurvival proteins were investigated *in vitro*. After preconditioning with 75% helium, neon, argon, krypton and xenon, cell cultures were subjected to OGD. Surprisingly, only xenon treatment showed protection of cell viability. Further, prosurvival proteins (Bcl-2, pAkt -Phospho-Akt- and HIF-1α-hypoxia inducible factor 1 α) were analyzed without OGD. Expression of HIF-1 α increased after treatment with argon, while Bcl-2 and p-Akt expression were not modified. However, xenon treatment led to an increase of all the examined proteins, Bcl-2, p-Akt and HIF-1α [10].

This is in contrast to the results mentioned above and may be due to different experimental settings (*in vivo* *vs*. *in vitro*), different models of tissue stress (hypoxic ischemic brain injury *vs*. naïve cell culture) and different time points of analysis.

Multiple damaging agents were tested in an *in vitro* study using a human osteosarcoma cell line (U2OS). Cells were exposed to a tyrosine kinase inhibitor (staurosporine), a DNA-damaging agent (mitoxantrone) and mitochondrial toxins. Argon and xenon inhibited cell loss by staurosporine, mitoxantrone and the mitochondrial toxins, maintained mitochondrial integrity and inhibited caspase-3 expression [43]. Suppression of caspase-3 and cytochrome C once again indicates inhibition of intrinsic apoptotic pathway by argon and xenon.

Using microglial cell cultures and primary neuronal and astroglial cultures the involvement of ERK1/2 (extracellular signal-regulated kinases) with a short and enhanced activation after exposure to 50% argon was demonstrated, but no relevant influence on cytokine expression (contrary to xenon) was found [38].

Finally, protein interactions of argon have to be mentioned: Colloch’h and colleagues investigated the protein-noble gas interactions of xenon, krypton and argon [48]. Three different enzymes were studied showing gas occupancies in the order of their polarizability with highest occupancy reached by xenon and lowest by argon administration, which is similar to the results of Quillin and colleagues examining T4 lysozyme [49]. Depending on the enzyme, different mechanisms of noble gas action were demonstrated: either inhibition of the catalytic reaction through an indirect mechanism, inhibition of the catalytic reaction through a direct mechanism, or prevention of substrate binding. The considerable effects of noble gases are not completely explained by the binding through very weak non-covalent van der Waals interactions. Therefore, the authors conclude that small effects on an array of biological targets may be responsible for the biological effects of noble gases but specific effects (like neuroprotection) of the noble gases may also be due to action via one particular target, which may be specific for each noble gas [48].

In conclusion, argon may distinguish itself from xenon while possibly sharing some joint features during further signaling (like Bcl-2 involvement). Also ERK1/2-signaling plays a role in signal transduction by argon. Decidedly, argon seems not to act via NMDA-receptor signaling or via potassium channels. Although argon would act as a GABA_A_ agonist to induce narcosis as shown in hyperbaric conditions, whether this could apply to normobaric condition as a mechanisms for neuroprotection still remains to be shown. Therefore, the precise target(s) for the biological effects generated by argon administration remains to be elucidated. Only limited evidence indicates the involvement of GABA_A_-receptor signaling. Finally, approaching the topic from the chemical point of view, one has to highlight the assessment of two important chemists (Nikolai Nikolajewitsch Semjonow and Cyril Norman Hinshelwood), who pointed out the oxygen-like properties of argon: the presence of argon allows reactions between phosphorous and oxygen under pressure levels, which would not happen without argon, therefore acting as sort of catalyst [50]. Thus, increase of resistance towards hypoxia may be explained by argon’s oxygen-like properties as hypothesized by David and colleagues previously [11,36].

### 3.4. Lack of Clarity

However, while appreciating many promising details of argons possible protective actions, some discrepancies should not be overlooked:

*In one in vivo study under hypoxic argon atmosphere, mice did not survive longer than the control group [9]. Another in vitro study using OGD as experimental model did not disclose a beneficial effect of argon preconditioning [10]. Finally, argon treatment in rats applying MCAO resulted in one study in reduced infarct volume (including subcortical area) but in the other in increased infarct volume of subcortical area and worse neurological outcome [11,34]. During one trial the application of argon occurred within the intraischemic phase, and on another occasion after reperfusion, as David and colleagues clearly pointed out [11].* 

These discrepancies may be attributed to differences in the study protocol. One major problem analyzing the studies on argon is that treatment varies between pre-conditioning and post-conditioning. Even if the same “type” of treatment is applied, timing, concentration and duration of administration diverge.

Therefore, to gain more insights into argon’s protective effects as well as identifying its mechanisms of action, standardizing study protocols would be advantageous. Argon’s cytoprotective and special neuroprotective properties have been demonstrated in many studies. Transfer into clinics has not yet occurred due to a lack of data for argon’s practical implementation and potential side effects. David and colleagues [36] tested argon in the context of tPA (tissue-type plasminogen activator) application to review a potential application in stroke therapy. Results demonstrate a dual argon effect. The somehow unexpected inhibiting effect of argon at low concentration on tPA efficiency according to the authors may be due to aforementioned interactions with proteins dependent on multiple factors like gas accessibility and affinity to hydrophobic cavities and the oxygen-like properties of argon [36]. Thus, additionally considering dual effects is necessary for further identification of the appropriate clinical administration concerning timing and duration as well as detection of the mechanism of action.

## 4. Methods

A PubMed search was carried out in June 2014 with the following search terms: neuroprotection OR organ protection OR cell death OR neuro* OR hypoxic ischemic encephalopathy OR asphyxia OR ischemia OR hypoxia OR ogd OR tbi OR protect* AND argon. Additionally, alternative databases (Embase, Scisearch, Biosys, gms) were screened for the same search terms. Afterwards duplicates were eliminated. The reference lists of review and other relevant articles were hand-searched for appropriate articles and two additional articles, which were later published online ahead of print, were included as well. Of note, Russian articles have been translated by a non-native speaker and therefore we might have caused a translation bias. Additionally, the heterogeneity of experimental settings may hinder the final appraisal.

## 5. Conclusions

Argon’s neuroprotective and organoprotective properties have been demonstrated repeatedly, but still uncertainties arise from the inhomogeneity of applied models, timing and dosage of argon application.

## Appendix

Members of the AON group:

Anne Brücken, University Hospital RWTH Aachen, Aachen, Germany

Michael Fries, University Hospital RWTH Aachen, Aachen, Germany

Oliver Kepp, INSERM, U848, Villejuif, France

Marc Lemaire, Air Liquide Santé, Paris, France

Daqing Ma, Imperial College London, UK

Guy Magalon, Hospital CHU Timone, Marseille, France

Patrick P. Michel, Inserm U 1127, CNRS UMR 7225, Sorbonne Universités, UPMC Univ Paris 06 UMR S 1127, Institut du Cerveau et de la Moelle Epinière, Paris, France

Arne Neyrinck, UZ Leuven, Leuven, Belgium

Jan Pype, Air Liquide Santé, Paris, France

Steffen Rex, UZ Leuven, Leuven, Belgium

Robert D. Sanders, University College London, London, UK

Sinead Savage, Imperial College, London, UK

Christian Stoppe, University RWTH Aachen, Aachen, Gemany
